# HDAC Inhibitor Titration of Transcription and Axolotl Tail Regeneration

**DOI:** 10.3389/fcell.2021.767377

**Published:** 2021-12-31

**Authors:** S. Randal Voss, Jeramiah J. Smith, Raissa F. Cecil, Mirindi Kabangu, Timothy J. Duerr, James R. Monaghan, Nataliya Timoshevskaya, Larissa V. Ponomareva, Jon S. Thorson, Alan Veliz-Cuba, David Murrugarra

**Affiliations:** ^1^ Department of Neuroscience, Spinal Cord and Brain Injury Research Center, and Ambystoma Genetic Stock Center, University of Kentucky, Lexington, KY, United States; ^2^ Department of Biology, University of Kentucky, Lexington, KY, United States; ^3^ Department of Biology and Institute for Chemical Imaging of Living Systems, Northeastern University, Boston, MA, United States; ^4^ College of Pharmacy and Center for Pharmaceutical Research and Innovation, University of Kentucky, Lexington, KY, United States; ^5^ Department of Mathematics, University of Dayton, Dayton, OH, United States; ^6^ Department of Mathematics, University of Kentucky, Lexington, KY, United States

**Keywords:** HDAC, axolotl (*Ambystoma mexicanum*), regeneration, romidepsin, transcription, single nuclei RNA-seq, CRISPR-Cas9

## Abstract

New patterns of gene expression are enacted and regulated during tissue regeneration. Histone deacetylases (HDACs) regulate gene expression by removing acetylated lysine residues from histones and proteins that function directly or indirectly in transcriptional regulation. Previously we showed that romidepsin, an FDA-approved HDAC inhibitor, potently blocks axolotl embryo tail regeneration by altering initial transcriptional responses to injury. Here, we report on the concentration-dependent effect of romidepsin on transcription and regeneration outcome, introducing an experimental and conceptual framework for investigating small molecule mechanisms of action. A range of romidepsin concentrations (0–10 μM) were administered from 0 to 6 or 0 to 12 h post amputation (HPA) and distal tail tip tissue was collected for gene expression analysis. Above a threshold concentration, romidepsin potently inhibited regeneration. Sigmoidal and biphasic transcription response curve modeling identified genes with inflection points aligning to the threshold concentration defining regenerative failure verses success. Regeneration inhibitory concentrations of romidepsin increased and decreased the expression of key genes. Genes that associate with oxidative stress, negative regulation of cell signaling, negative regulation of cell cycle progression, and cellular differentiation were increased, while genes that are typically up-regulated during appendage regeneration were decreased, including genes expressed by fibroblast-like progenitor cells. Using single-nuclei RNA-Seq at 6 HPA, we found that key genes were altered by romidepin in the same direction across multiple cell types. Our results implicate HDAC activity as a transcriptional mechanism that operates across cell types to regulate the alternative expression of genes that associate with regenerative success versus failure outcomes.

## Introduction

Transcription differs within and between cell types and varies in response to extrinsic and intrinsic cues, such as when cells are challenged by pathogens or when cells respond to signaling molecules during development. Analyses of transcription can therefore reveal the identities and phenotypes of cells, and how genes function and interact to regulate biological processes. It is standard to perturb gene and protein functions and then use transcriptional analysis to identify key changes in molecular and cellular states that are informative for understanding biological mechanisms. For example, gene knock-out and knock-in technologies can be used to decrease or increase the expression of specific transcription factors to identify downstream target genes and the composition of gene regulatory networks (Schenone et al., 2013; Varshney et al., 2015). Alternatively, small molecules can be used to specifically alter the activities of regulatory proteins to interrogate mechanisms of transcriptional regulation (Yeh and Crews, 2003; Arrowsmith et al., 2012).

Amphibians like the laboratory axolotl (*Ambystoma mexicanum*) are capable of regenerating whole organs in aquatic environments that facilitate rapid screening of small molecules (Ponomareva et al., 2015). To advance regeneration research, we evaluate here an experimental approach to detail chemical effects on transcription and regeneration. Regeneration requires numerous changes in gene expression from the moment of injury to the time a tissue is completely repaired. Each gene thus provides a transcriptional biomarker that can be used to detail a chemical’s effect on tissue regeneration. A transcriptomic approach is potentially made more powerful by conceptualizing regeneration as a discrete trait, with definable regenerative failure vs regenerative success outcomes. We propose that for some chemicals there is a critical concentration; above and below this threshold, regeneration will either fail or succeed. Thus, by quantifying transcription at concentrations that span a chemical’s critical threshold concentration, it might be possible to identify quantitative changes in key genes that determine regeneration outcome, and through subsequent experimental, computational and bioinformatic approaches, associate these quantitative changes to biological processes and properties of cell populations. We evaluate this approach using romidepsin (Ueda et al., 1994; Yang et al., 2011), an FDA approved histone deacetylase inhibitor that potently inhibits axolotl embryo tail regeneration. Application of this approach to additional chemicals offers potential to develop rich information resources that can be used to characterize and model chemical effects and gene interactions on tissue regeneration, identify promising chemical tools for regenerative biology, and identify chemical and biological mechanisms of action.

## Materials and Methods

### Animal Procedures

Non-feeding embryos used in this study were treated according to the same ethical standards that apply to feeding axolotls under University of Kentucky IACUC protocol 2017-2580. Embryos (RRID:AGSC_100E, AGSC_101E, AGSC_102E) were obtained from the Ambystoma Genetic Stock Center (RRID:SCR_006372) and all experiments were performed using axolotl rearing water (ARW: 1.75 g NaCl, 100 mg MgSO4, 50 mg CaCl2, and 25 mg KCl per liter, buffered with NaHCO_3_ to pH 7.3–7.5) in a room maintained at 17–18°C.

### Romidepsin Dosing Experiments

Developmental stage 42 (Bordzilovskaya et al., 1989) axolotl embryos were manually hatched by removing the egg jelly and membrane, anesthetized in 0.02% benzocaine, and tail amputations were performed with a sterile razor blade to remove 2 mm (∼20% of the body length) of the distal tail tip. Axolotl embryos were then distributed into 12-well microtiter plates containing romidepsin or axolotl rearing water (ARW) with DMSO. Romidepsin (Selleckchem, Cat. No. S3020) was dissolved in DMSO and diluted to a stock concentration of 10 mM. The romidepsin stock solution was subsequently diluted to a range of concentrations (0, 0.05, 0.1, 0.5, 1.0, 5.0, and 10.0 μM) and 2 replicates of 3-6 embryos were treated for 6 or 12 h per concentration, and embryos were imaged at 6 days post-amputation (DPA) using an Olympus dissecting microscope with ×0.5 objective lens and DP400 camera. These initial concentration experiments were performed to identify the critical concentration, above and below which regeneration succeeds or fails. Distal tail shape was used to classify concentrations as inhibitory or having no effect on tail regeneration at 6 DPA (Voss et al., 2019).

### Romidepsin Treatment and Transcription

Embryos were administered tail amputations and treated with the same range of concentrations of romidepsin (0, 0.05, 0.1, 0.5, 1.0, 5.0, and 10 μM) as described above, for 6 and 12 h post amputation (HPA). One mm of distal tail tip tissue was collected from each embryo within a replicate and pooled for RNA isolation using Trizol followed by Qiagen miniprep. Overall, 60 samples were processed. Four replicates (12 embryos each) were performed for each romidepsin concentration and treatment time; three replicates were performed for the control sample at the time of amputation. A total of 100 genes (Supplementary Table S1) were selected from previous studies of axolotl embryo tail regeneration (Ponomareva et al., 2015; Voss et al., 2019) to develop a Nanostring probeset for quantifying transcript number. Most of these genes (N = 72) were shown previously to be differentially expressed in response to romidepsin treatment (Voss et al., 2019). RNA samples were processed by the University of Kentucky Healthcare Genomics Core. Transcript data were normalized using nSolver software and mRNA count data from low, moderate, and highly expressed genes that presented low coefficients of variation for transcript number across treatments. The Nanostring probesets are presented in Supplementary Table S1, the normalized transcript count data in Supplementary Table S2, and Benjamini and Hochberg (1995) corrected *p*-values (considered significant if < 0.1) are presented in Supplementary Table S3.

### Computational Modeling of Romidepsin Transcriptional Dose Responses

Transcript abundance estimates across romidepsin concentrations yielded response curves for all 100 genes at 6 and 12 HPA. Non-linear modeling Was performed to identify genes with sigmoidal or biphasic response Curves. The sigmoidal model

$$\left(a+\left(b-a\right)\right)/\left(1+e^{\left(-k\left(x-th\right)\right)}\right)$$

used four parameters: the minimum transcript number **(a)**, the maximum transcript number **(b)**, the concentration of romidepsin that yielded a transcript abundance halfway between concentrations that defined minimum and maximum transcription outputs **(th)**, and a parameter controlling the slope **(k)**. The biphasic model used the product (and the sum) of two sigmoidal functions. Two types of errors were considered in classifying genes into these categories: 1) the least square error and 2) the least square error divided by the range (maximum response–minimum response). Response curves with scaled errors less than 0.4 were classified as sigmoidal. Genes that did not meet the sigmoidal error criteria but presented response curves with scaled errors less than 0.4 were classified as biphasic. The modeling results are presented in Supplementary Table S5.

### Genetic and Chemical Inhibition of Hyaluronic Synthase 2

The functional role of *Has2* in axolotl embryo tail regeneration was evaluated by CRISPR-Cas9 gene editing and chemical inhibition using calcitriol. First, tw guide RNAs (TGG​CTA​CCA​ATT​CAT​CCA​GA; GCT​CGT​CCT​CTC​CAA​CAA​GT) were designed against *Has2* protein-coding sequence and two target-specific Alt-R crRNAs and common Alt-R tracrRNA were synthesized by Integrated DNA Technologies (Amex_G.v6 genome assembly HAS2|AMEX60DD301040413.1). Alt-R–Cas9 Ribonucleoprotein complexes for both guide RNAs were prepared and injected into 1-cell stage axolotl embryos as described previously (Trofka et al., 2021). Thirty-two injected and 10 non-injected control embryos were reared to developmental stage 42 and tail tips were amputated as described above. Tail tips from 6 injected embryos were used to test for CRISPR-Cas9 gene editing by DNA isolation (Monarch Genomic DNA Isolation Kit), PCR (Forward Primer: 5-AAA​TAG​TCT​GGC​AGA​TTC​CAA​TTC-3; Reverse Primer: 5-CAT​TCA​TGA​ACA​GAC​TGA​AAG​GAG-3) and DNA sequencing (Eurofins). PCR was performed usin 34 cycles (95°C 45 s, 60°C 45 s, and 72°C 30 s) and an Applied Biosystems Veriti 96-well thermocycler. PCR products were prepared for sequencing using Exo-Cip (New England Biolabs). At 7 DPA, the amount of tail tip tissue regenerated was quantified from images obtained using the Olympus microscope and camera described above, using the polyline tool in the Olympus cellSens standard 1.5 imaging software program to outline the area of the tail between the amputation plane and distal tail tip. Second, tail tips of developmental stage 42 embryos were amputated, and embryos were treated with *Has2* inhibitor calcitriol (Narvaez et al., 2020), the active form of vitamin D. Calcitriol was purchased from Selleckchem.com and diluted to 10 mM using DMSO. Four embryos (per each concentration tested) were treated using 0.0, 0.1, 0.25, 0.5, 0.75, and 1.0 μM and reared to 7 DPA for imaging of the amount of tissue regenerated. The amount of tissue regenerated was quantified as described above.

### Whole Mount Version 3 Hybridization Chain Reaction Fluorescent *In Situ* Hybridization

The protocol outlined below is based off protocols provided by Molecular Instruments. Tissues were collected and fixed in 4% PFA overnight at 4°C. The following day, the tissues were washed three times for 5 min at room temperature with PBST (1X PBS with 0.1% Tween-20). The tissues were dehydrated in an increasing methanol series (25% MeOH/75% PBST, 50% MeOH/50% PBST, 75% MeOH/25% PBST) on ice for 5 min at each step and placed in 100% MeOH at −20°C overnight. At this point, the tissue could be left at −20°C indefinitely. Tissues were rehydrated in a decreasing methanol series (75% MeOH/25% PBST, 50% MeOH/50% PBST, 25% MeOH/75% PBST) on ice for 5 min at each step and washed in PBST for 5 min at room temperature. To remove pigments, samples were bleached in 3% H_2_O_2_ (made in 0.8% KOH) for an hour at room temperature. Samples were next washed in PBST three times for 5 min at room temperature. Tissues were washed in pre-warmed hybridization solution (Molecular Instruments, https://www.molecularinstruments.com) for 5 min at 37°C. This hybridization solution was replaced with fresh, pre-warmed hybridization, and samples were incubated at 37°C for 30 min. Probe solution was made by diluting 1 µM probe stock 1:200 in hybridization solution. Probe sequences are provided in Supplementary Table S4. The probe solution was then applied to the samples and incubated at 37°C overnight. The next day, the samples were washed four times for 15 min with pre-warmed probe wash (Molecular Instruments) at 37°C. Samples were next washed t in 5X SSCT (5X SSC with 0.% Tween-20) for 5 min at room temperature. Following these washes, samples were incubated in amplification buffer (Molecular Instruments) for 30 min at room temperature. As samples are incubating in amplification buffer, fluorescent hairpins (Molecular Instruments) were incubated at 95°C for 90 s, then left to return to room temperature for minimally 30 min. Hairpins were diluted 1:50 in amplification buffer, and this hairpin solution was applied to the samples and incubated at room temperature overnight. The next day, samples were washed twice with 5X SSCT at room temperature for 30 min each. For imaging with light sheet fluorescence microscopy, samples were mounted in 1.5% low melt agarose in a capillary tube. Agarose containing the samples were briefly washed in ×1 P for 10 min, then placed in Easy Index (Life Canvas Tech) overnight at 4°C. Samples were imaged at ×5 , and maximum intensity projections were used for display within the figures and quantification.

### V3 HCR-FISH Image Analysis

For quantification of V3 HCR FISH fluorescence intensity, we used custom FIJI (Schindelin et al., 2012) macros to measure the raw integrated density in 1 µm wide boxes along the AP axis of injured and uninjured tails as described previously (Duerr et al., 2021). Briefly, the tails were rotated such that the most posterior tip pointed to the right. Next, the tail outline was segmented, and the tip of the injured or uninjured tail was marked with a point. A 1 µm wide box that extended to the dorsal and ventral fins was then created anterior to this point, and the raw integrated density was measured within this box and in boxes extending 500 µm from the posterior tip. The raw integrated density was normalized to the area within the boxes and plotted to observe differences in intensity between injured and uninjured tails.

### Single Nuclei RNA-Seq

To map transcripts to axolotl cell types, single-nuclei RNA-Seq was performed. Embryos were administered 2 mm distal tail amputations and either treated in ARW (N = 100) or 10 μM romidepsin (N = 100). At 6 HPA, 1 mm of distal tail tip tissue was collected and pooled for nuclei isolation and ×10 single nuclei RNA-Seq. Nuclei isolation, library preparation, and next generation sequencing were performed by Singulomics. The resulting data were mapped to an axolotl transcript assembly as described previously (Rodgers et al., 2020), analyzed using Cell Ranger, and visualized using ×10 visualization software (Loupe version 5.0). Default graph-based clustering was used to identify distinct clusters of cells and enriched genes were used to manually identify and differentiate among cell types. The single nuclei RNA-Seq data were submitted to NCBI Gene Expression Omnibus for public release upon publication.

## Results

### A Critical Romidepsin Dose Defines Regenerative Outcome

**FIGURE 1:**
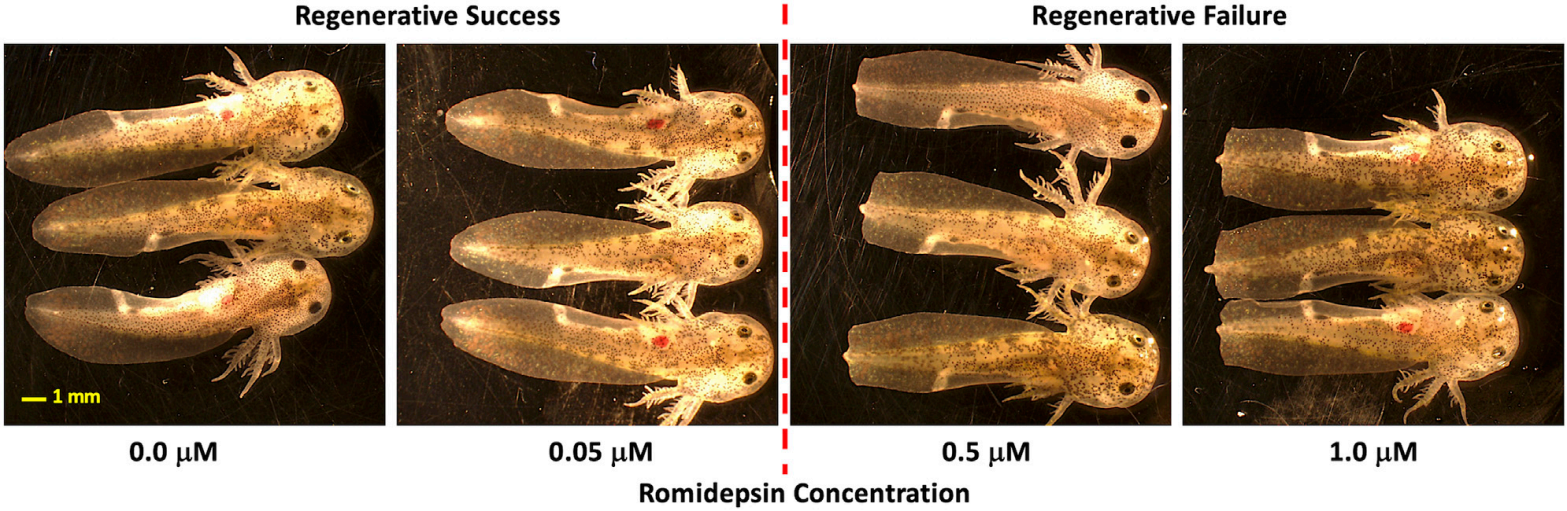
Concentration dependent effect of romidepsin on tail regeneration. Embryos that were treated continuously for 6 and 12 HPA with ≤0.05 μm romidepsin fully regenerated their tails while embryos treated with ≥0.5 μm romidepsin presented blunt-shaped tails (a non-regenerative outcome).

In previous experiments, we showed that 10 μM romidepsin, applied for 1-min post-amputation or longer, inhibits axolotl embryo tail regeneration at 6 DPA (Voss et al., 2019). We therefore treated embryos with lower concentrations of romidepsin to identify a critical concentration that reproducibly defined alternative regeneration success versus failure outcomes. Embryos that were treated continuously for 6 and 12 HPA with ≤0.05 μM romidepsin fully regenerated their tails while embryos treated with ≥0.5 μM romidepsin presented blunt-shaped tails consistent with a non-regenerative outcome (Figure 1). Thus, the critical concentration for regenerative success and failure outcomes was defined as ≥ 0.05 and ≤0.5 μM romidepsin. We note that if embryos are treated for only 1 min post amputation, the critical concentration defining alternative regeneration outcome is higher (≥0.5 and ≤1.0). Thus, the critical concentration for romidepsin and likely other chemicals, depends upon both concentration and dosage time.

### Repeatability of the Effect of Romidepsin on Transcription

We next performed a transcriptional analysis of 100 genes across a range of romidepsin concentrations (0, 0.1, 0.05, 0.5, 1.0, 5.0, 10.0 μM). Seventy-two of the genes in this set (Supplementary Table S3) were previously shown by microarray analysis to be differentially expressed (i.e., significantly different when comparing romidepsin treated and untreated embryos) at either 6 or 12 HPA in response to 10 μM romidepsin (Voss et al., 2019). Of these 72, 53 were identified in this study as differentially expressed at either 6 or 12 HPA in response to 10 μM romidepsin. For the remaining19 genes, all but 5 yielded a significant *p*-value for one or more of the <10 μM romidepsin concentrations that were tested in this study. Overall, these results show that romidepsin provides a reproducible chemical tool for investigating transcription.

**FIGURE 2:**
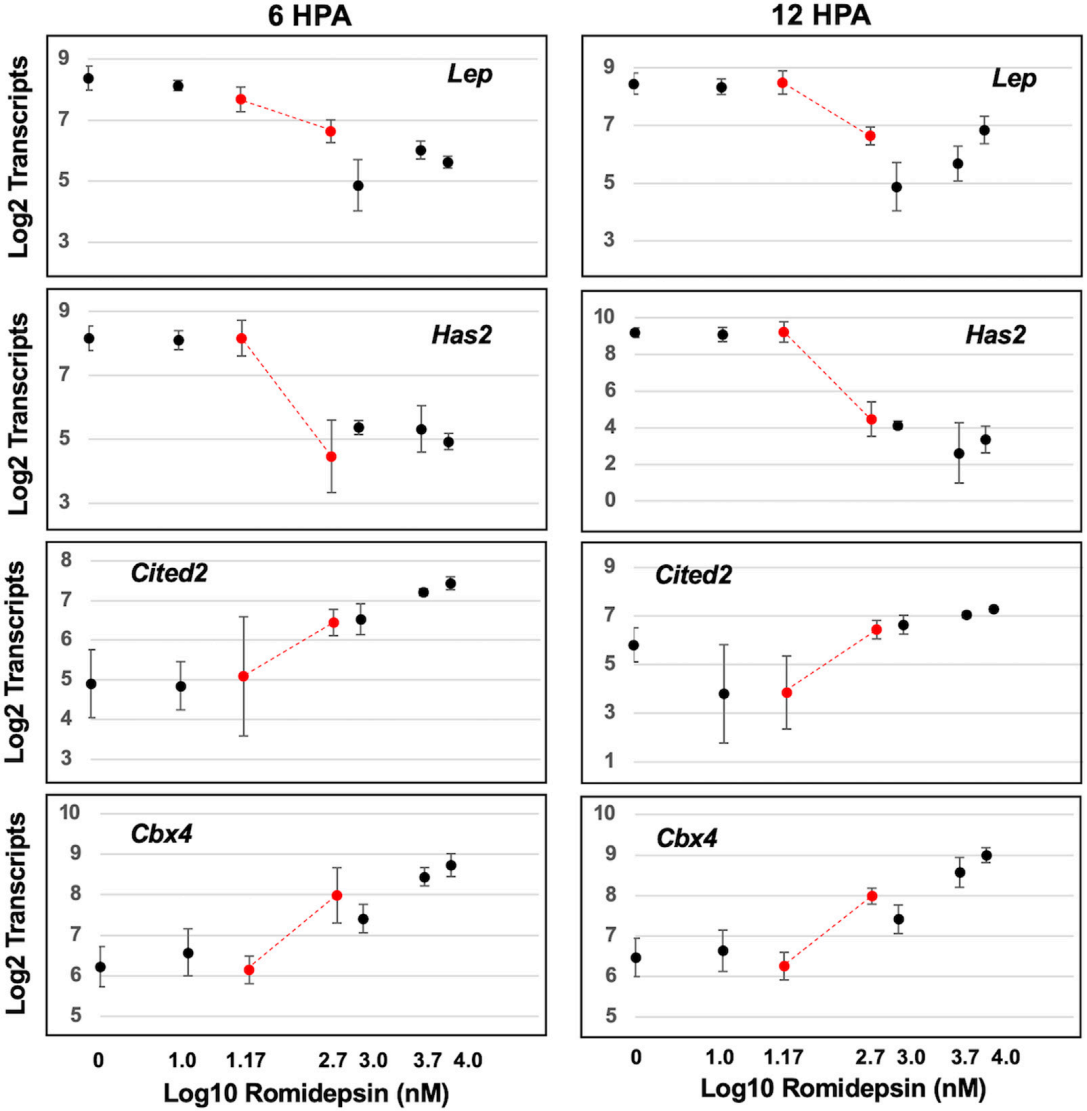
Concentration dependent effect of romidepsin on transcription. Examples of four genes that were classified as exhibiting sigmoidal transcript response curves. *Has2 and Lep* transcripts decreased within increasing romidepsin concentration. *Cited2* and *Cbx4* transcripts increased with increasing romidepsin concentration. The red dots correspond to romidepsin concentrations that were associated with regenerative (0.05 μM) and non-regenerative outcomes (0.5 μM).

We next examined transcript abundances as a function of romidepsin concentration. We sought to identify transcript response curves that changed prior to and within the critical concentration of romidepsin that determined regenerative outcome. Concentration-response relationships typically follow a monotonic sigmoidal function although more complicated, biphasic functions are also possible (Calabrese, 2013). Thus, we performed non-linear modeling to identify genes with sigmoidal or biphasic response curves (Supplementary Table S5). Non-linear changes in transcript abundance were observed, with some genes presenting significantly lower transcript abundances at regeneration inhibitory versus permissive concentrations of romidepsin, and others showing the opposite pattern (Figure 2). Previously, the expression of *Cited2* and *Cbx4* was shown to be significantly up-regulated by 10 μM romidepsin and *Has2* and *Lep* were shown to be significantly down-regulated (Voss et al., 2019). *Lep* and *Has2* are expressed in fibroblast-like progenitor cells (Leigh et al., 2018; Rogers et al., 2020) and therefore might be required for regeneration while *Cited2* is up-regulated under conditions of cellular stress and regenerative failure (Baddar et al., 2021). Here, by varying romidepsin concentration, we show that transcriptional output at these and other loci is concentration dependent. *Cbx4* and *Cited2* presented monotonically increasing transcriptional responses while *Has2* and *Lep* presented monotonically decreasing responses. Overall, 90 of 100 genes were classified as biphasic or sigmoidal at either 6 or 12 HPA, and 38 transcription response curves had inflexion points between 0.05 and 0.5 μM romidepsin (Supplementary Table S5). Transcriptional output for the majority of genes targeted in this study was dose-dependently affected by romidepsin and presumably, quantitative changes in HDAC activity.

### Functional Analysis of Has2

**FIGURE 3:**
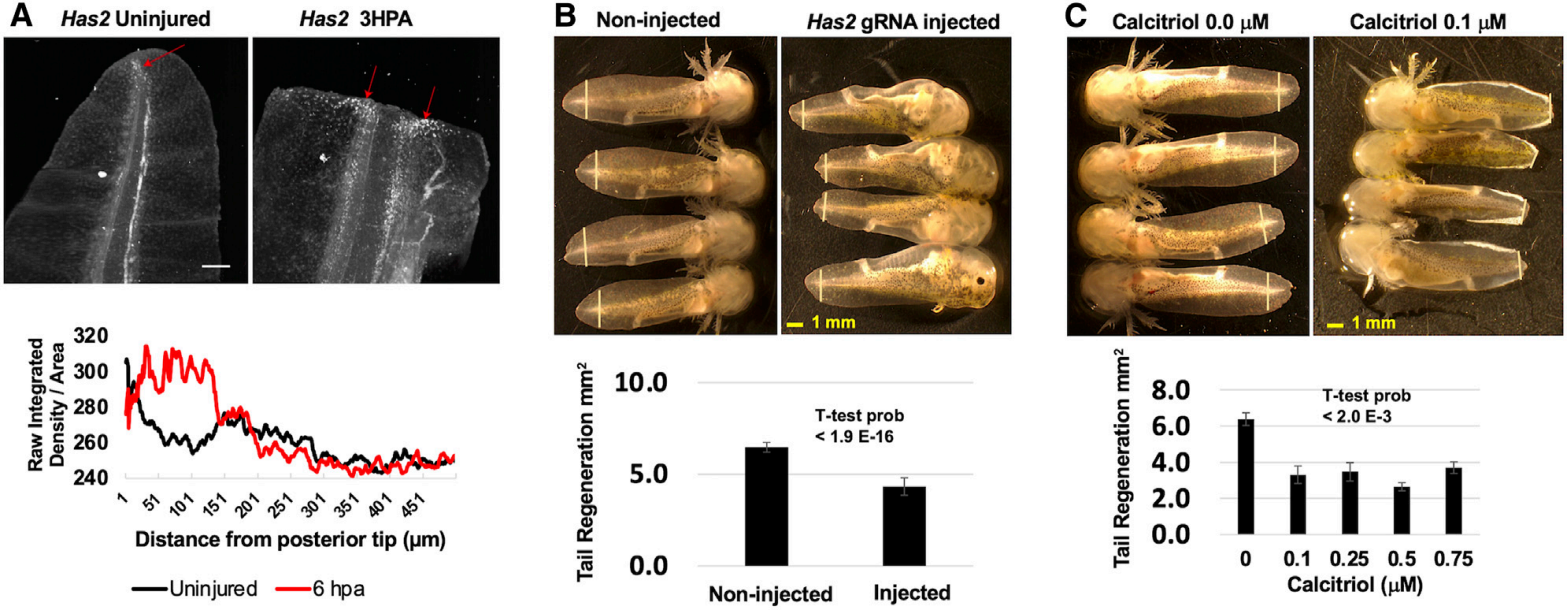
Functional analysis of *Has2*. **(A)** *Has2* expression increased after tail amputation and was highest near the middle of the tail and at the amputation plane where the blastema subsequently forms. The red arrows indicate *Has2* positive cells and the scale bar is 250 μm. **(B)** CRISPR-Cas9 and gRNAs targeting *Has2* were injected into embryos. Injected and non-injected embryos were reared to developmental stage 42 and tails were amputated. Injected embryos presented enlarged hearts, edema, and little to know vasculature. At 7 DPA, on-injected embryos regenerated significantly more tail tissue than injected embryos. The yellow vertical lines indicate the plane of amputation. **(C)** Developmental stage 42 embryos were administered DMSO or hyaluronan synthase inhibitor calcitriol after tail amputation. Calcitriol-treated embryos presented white patches on their gills and tail fins. At 7 DPA, DMSO-treated embryos regenerated significantly more tail tissue than calcitriol-treated embryos. The yellow vertical lines indicate the plane of amputation.

Several of the genes that were identified as romidepsin-dose dependent have previously been identified as differentially expressed in axolotl tissue regeneration studies, but none of the genes have been tested functionally. To assess function, we focused on *Has2,* as hyaluronan synthesis is required for zebrafish fin (Ouyang et al., 2017) and Xenopus tail regeneration (Contreras et al., 2009). *Has2* is expressed by blastema-like progenitor cells in the regenerating axolotl limb (Leigh et al., 2018; Rodgers et al., 2020) and we similarly observed an increase in *Has2* expression along the amputation plane where the tail blastema forms during regeneration (Figure 3A). To determine if *Has2* is also required for axolotl tail regeneration, we knocked down *Has2* using genetic and pharmaceutical approaches. First, we performed CRISPR-Cas9 injections, injecting two gRNAs for *Has2* coding sequence into 1-cell stage embryos. The resulting embryos were reared to developmental stage 42 and tail tips were amputated. During regeneration, all but two injected embryos (N = 32) presented pericardial edema, enlarged irregularly beating hearts, and little to no peripheral vasculature; similar phenotypes were described previously for *Has2* knock-out mice (Camenisch et al., 2000). A sample of embryos (N = 6) presenting edema and vascular defects were confirmed to have *Has2* genome edited alleles (Supplementary Figure S1). Interestingly, *Has2* embryos regenerated tail tissue, but the overall amount was significantly less than observed for non-injected embryos (Figure 3B). To complement the genetic knock-down approach, a separate group of embryos were reared to developmental stage 42, tail tips were amputated, and embryos were administered different concentrations of calcitriol, an inhibitor of *Has2* expression (Narvaez et al., 2020). Calcitriol, the active form of vitamin D, increases calcium uptake and we observed a milky white substance in the gill tips and epithelia of treated embryos. At 7 DPA, calcitriol treated embryos regenerated significantly less tissue than controls (Figure 3C). These results suggest a requirement for *Has2* in axolotl tail regeneration, although additional studies are needed to determine if the approaches used to knock-down *Has2* function affected regeneration directly or indirectly.

### Single-Nuclei Analysis of Distal Tail Cells at 6 HPA

**FIGURE 4:**
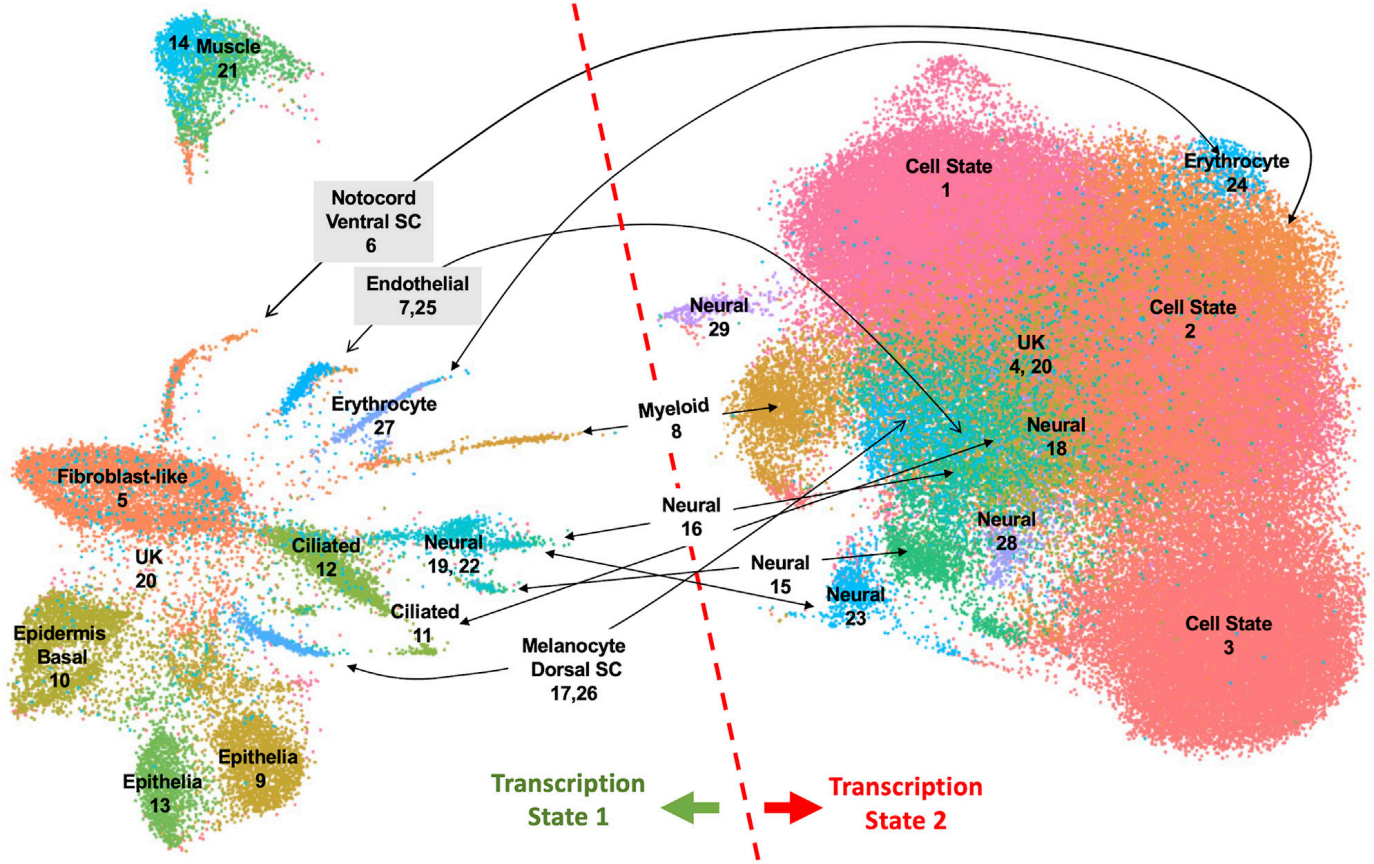
UMAP projection of 133,193 nuclei isolated from axolotl embryo tail tips and characterized by single nuclei RNA-Seq. Twenty-nine clusters were identified from an analysis of nuclei isolated from control 0 HPA, 6 HPA, and romidepsin 6 HPA embryos. Clusters were annotated to cell types when possible. Nuclei in the left half of the UMAP projection expressed repetitive sequence transcripts and chromatin-modifying factors more highly than nuclei in the right half.

**FIGURE 5:**
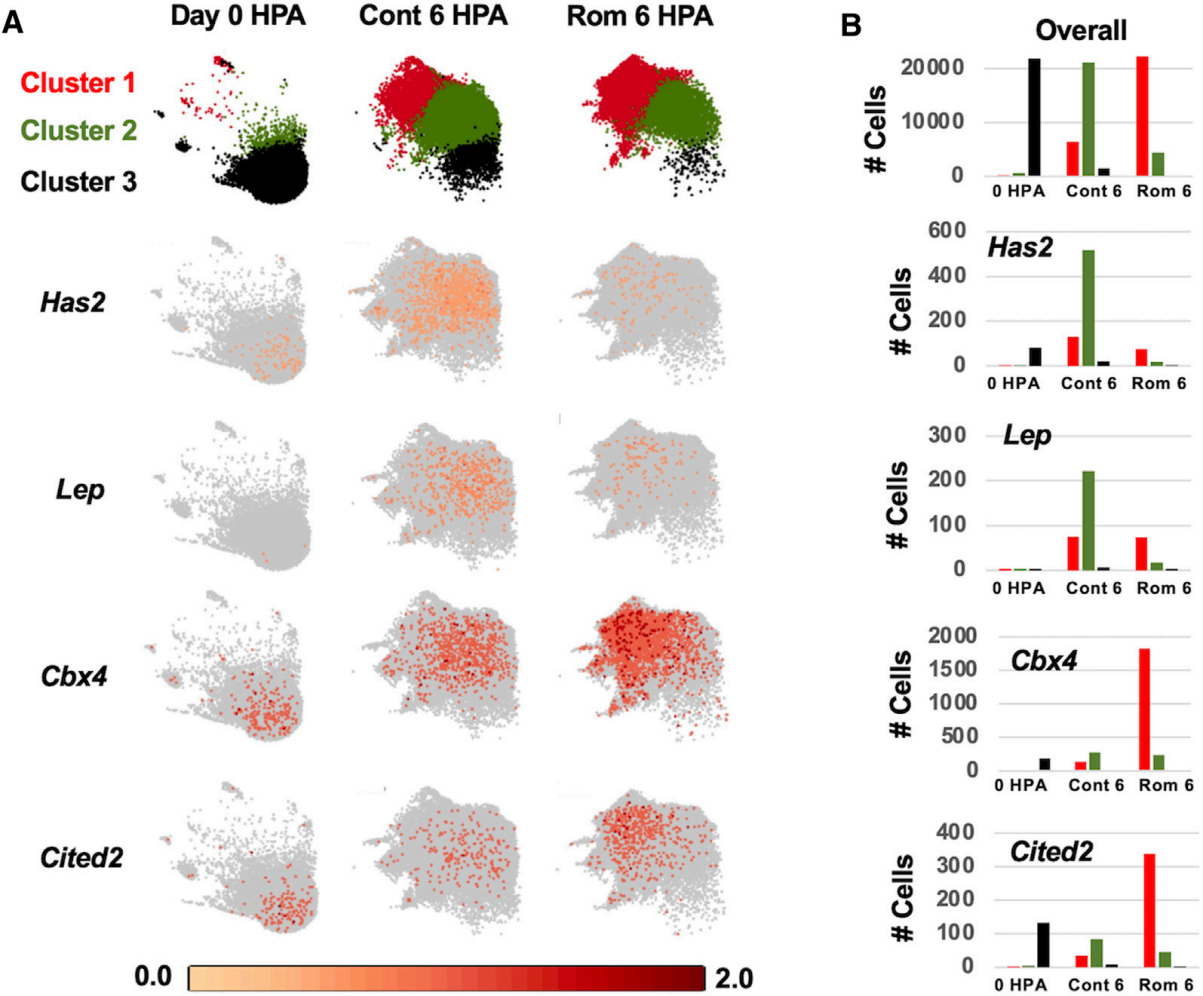
Log_2_ expression of regeneration associated genes among control 0 HPA, control 6 HPA, and romidepsin 6 HPA samples for clusters 1–3. **(A)** At 0 HPA, the majority of cells were observed in cluster 3 (black). At 6 HPA, there were few cluster 3 cells and the proportion of cluster 1 (red) and cluster 2 (green) cells varied between the control and romidepsin-treated samples. *Has2* and *Lep* were expressed more highly in Cont 6 HPA while *Cbx4* and *Cited2* were expressed more highly in Rom 6 HPA. **(B)** Number of cluster 1-3 cells expressing *Has2*, *Lep, Cbx4*, and *Cited2* among samples.

To investigate properties of romidepsin-moderated genes at the cellular level, we performed RNA-Seq of single nuclei isolated from amputated axolotl tail tips at the time of amputation (0 HPA, N = 31,522), and at 6 HPA in 10 μM romidepsin-treated (Rom 6 HPA, N = 56,936) and untreated embryos (Cont 6 HPA, N = 44,735). Considering all data, graph-based clustering identified 29 clusters with >309 nuclei (cells) in each cluster (Figure 4). Using genes that were expressed more highly within individual clusters relative to all other clusters, and Panther gene expression tools (Huaiyu et al., 2019) to identify enriched gene ontologies, cell types were annotated to clusters (Supplementary Table S6). Cell types typical of embryonic tail tissues were identified, including epidermal, epithelial, muscle, fibroblast, notochord, spinal cord, endothelial, erythrocyte, and multiple neural cell types. However, the three largest clusters (1–3, N = 79,533 nuclei) did not present genes that were characteristic of any single differentiated cell type, and thus are likely comprised of multiple cell types. For example, genes identified as enriched in muscle (*Rrad*), erythrocytes (*Visg1, Alas2*), and fibroblasts (*Has2, Lep*) were enriched in cluster 2, while genes associated with the regulation of general biological processes, including transcriptional regulation (*Cbx4, Hoxa1, Egr2, Cited2, Junb*), were enriched in cluster 1. Samples included in this study differed widely in their relative contribution to clusters 1–3. Considering all cells from clusters 1–3, 93% of cluster 3 cells were sampled by the 0 HPA library, 73% of cluster 2 cells were sampled by the Con 6 HPA library, and 83% of cluster 1 cells were sampled by the Rom 6 HPA library (Figure 5). We note that several of the genes that were upregulated by romidepsin in the Nanostring experiment (and associated with a non-regenerative outcome) were significantly upregulated in cluster 1 and not cluster 2, including *Cbx4*, *Cited2, Smad7, Spry1, and G0s2*. In contrast, cluster 2 contained regeneration-upregulated genes (*Lep, Has2*) that were down regulated by romidepsin in the Nanostring experiment. These data suggest an injury-associated transition of 0 HPA cluster 3 cells into 6 HPA injury states defined by clusters 1 and 2, with romidepsin driving a higher proportion of cells into a non-regenerative injury state defined by cluster 1.

**FIGURE 6:**
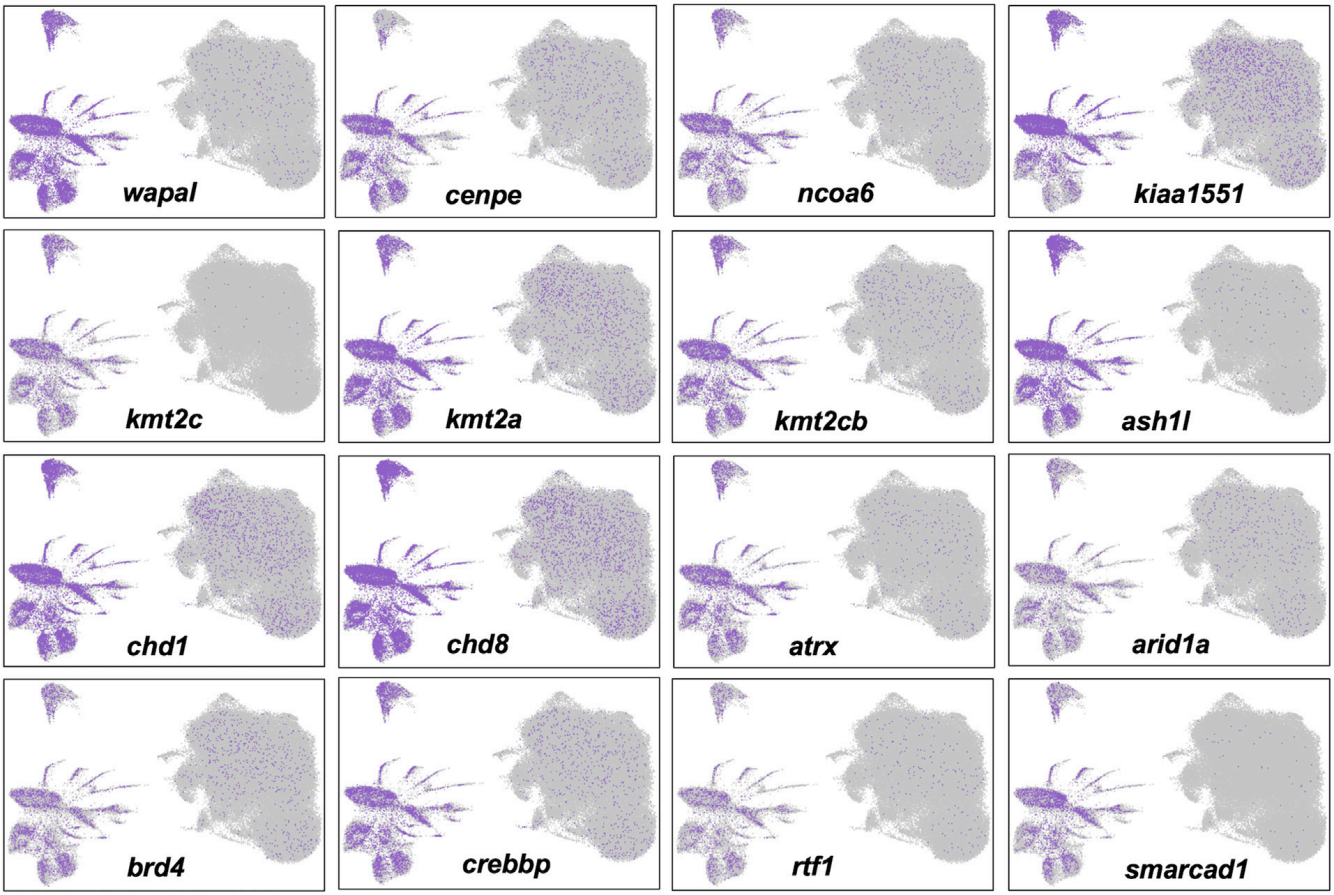
Alternative transcriptional states identified by single nuclei RNA-Seq are associated with the differential expression of epigenetic and transcription factors. Twelve genes that are known to function in histone modification, chromatin remodeling, and transcriptional regulation were more highly expressed in nuclei in the left half of the UMAP projection.

Cells in Clusters 1–3 grouped with cells from other clusters in the right half of the UMAP projection, to the exclusion of cells in clusters of the left half (Figure 4). These two different groups presented alternative transcriptional states defined by the relative expression of repetitive sequence-containing transcripts. Specifically, cells in the left half of the UMAP projection tended to express transcripts with repetitive sequences more highly than cells in the right half. We reasoned that these different transcriptional states might reflect a difference in global transcriptional output, with repetitive sequences passively reporting nascent transcription from loci distributed throughout the genome. In support of this hypothesis, we verified that transcripts reporting high levels of transcription contained repetitive elements and these elements were found to be distributed throughout the genome. We further reasoned that a global difference in transcriptional regulation may trace to chromatin modifying genes and indeed discovered many epigenetic and transcription factors whose transcription mirrored the expression of repetitive sequences (Figure 6). The high and low transcriptional states were identified within 0 HPA, Cont 6 HPA, and Rom 6 HPA libraries and thus cannot be attributed exclusively to injury or romidepsin, and they do not associate with the expression of typical cell cycle marker genes. Moreover, these alternative transcription states are not explained by spatial location as broad and even expression was observed throughout the uninjured and 3 HPA tail for one of the discriminating epigenetic factors (*Brd4*: Supplementary Figure S2). Further work will be needed to determine if the high and low transcriptional states identified in this study are a general characteristic of transcription from large axolotl genes and/or the capture of nascent and steady state transcripts by snRNA-Seq.

We next examined properties of cellular-level gene expression for 29 genes that were identified from the Nanostring experiment as significantly differentially expressed (*t*-test *p*-value < 0.01) at 6 HPA in response to 10 μM romidepsin, which was the concentration evaluated in the snRNA-Seq experiment. (Supplementary Table S7). The correlation of fold change was high between the platforms (r = 0.88); in other words, if a gene was expressed more highly in romidepsin treated vs. control embryos in the Nanostring experiment, fold change was also higher in Rom 6 HPA vs Cont 6 HPA (Supplementary Figure S3). Thus, gene expression for these 29 genes was similar at 10 μM romidepsin whether assayed at the tissue or nuclear level. We note that almost all of these genes presented >1.5 fold changes at romidepsin concentrations lower than 10 μM in comparison to baseline (0 μM romidepsin), with genes showing both positive and inverse dose dependency of transcription (Supplementary Figure S4).

**FIGURE 7:**
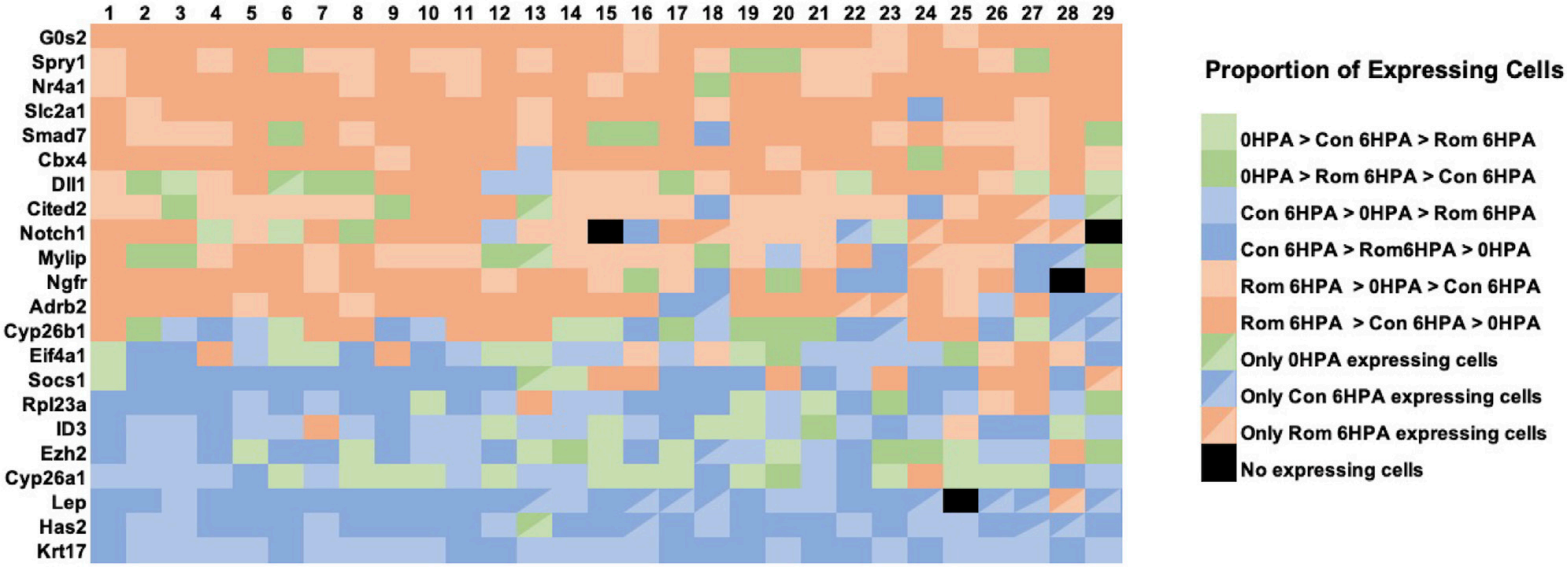
The relative proportion of cells within snRNA-Seq libraries and clusters that expressed transcripts for romidepsin-modulated genes. For each of 22 romidepsin-modified genes (see text), the proportion of expressing cells was determined for each library (Day 0, Cont 6 HPA, and Rom 6 HPA) and cluster (1-29) combination. Then, genes were classified for each cluster according to the rank ordering of expressing cells among the libraries. In the figure, sienna/tan colors indicate classifications where the highest proportion of expressing cells were observed in Rom 6 HPA, blue colors indicate classifications where the highest proportion of expressing cells were observed in Cont 6 HPA, and green colors indicate classifications where the highest proportion of expressing cells were observed in Day 0.

Several genes from the Nanostring experiment showed similar transcriptional responses to romidepsin. As described above, *Lep* and *Has2* response curves were sigmodal with high and low expression associated with regenerative success and failure outcomes, respectively. A correlated pattern of gene expression, detected at the tissue level, could reflect correlated changes in gene expression across a few or many cell types. To examine these possibilities, we determined the proportion of expressing cells for each library and cell type combination, again focusing on the 29 validated romidepsin-responsive genes. For this analysis, we conservatively required that a gene be expressed in greater than 5% of cells within at least 1 cell type; this filter eliminated 7 of the 29 genes. We then classified genes using rank ordering to show how they were expressed at the cellular level among the three snRNA-Seq libraries (Figure 7). For example, the proportion of expressing cells for a given cell type could be highest in the Rom 6 HPA library, next highest in the Cont 6 HPA library, and lowest in the 0 HPA library. This classification was most frequently observed for genes that were previously shown to be up regulated (e.g., *Cited2, Cbx4*) by 10 μM romidepsin, which was inhibitory to regeneration. Strikingly, this gene classification, or the next closest gene classification where the proportion of Rom 6 HPA cells was also highest overall (Rom 6 HPA >0 HPA > Cont 6 HPA), was observed across the majority of cell types. Alternatively, Cont 6 HPA > ROM 6 HPA > 0 HPA and Cont 6 HPA > 0 HPA > Rom 6 HPA classifications were more frequently observed for regeneration associated genes that were down regulated by 10 μM romidepsin, and again, these classifications were observed across cell types. We note that when the highest proportion of expressing cells was observed for the 0 HPA library, the classification 0 HPA > Rom 6 HPA > Cont 6 HPA was more frequent for genes up-regulated by 10 μM romidepsin while the classification 0 HPA > Cont 6 HPA > Rom 6 HPA was more frequent among genes that were down-regulated by 10 μM romidepsin. The non-random patterns observed in Figure 7 strongly suggest that key regeneration genes were not exclusively expressed by distinct cell types. Instead, transcriptional regulation appeared to be integrated across cell types by HDAC activity.

## Discussion

In this study we evaluated a method for detailing transcriptional changes that associate with alternative regeneration outcomes. We showed that romidepsin, a class I histone deacetylase inhibitor, provides a robust chemical tool for reproducible and dose-dependent alteration of transcriptional responses and regenerative outcome. We observed significant changes in transcription for genes at concentrations that were both permissive and inhibitory for tail regeneration, thus allowing us to identify genes that are most likely to be regulated by histone acetylation dynamics at the outset of regeneration. This approach may also help prioritize candidates for functional studies if transcriptionally modified genes are more likely to affect a successful regeneration outcome. In support of this hypothesis, we used genetic and pharmaceutical approaches to knock down *Has2* and generate data suggesting a requirement for *Has2* in axolotl embryo tail regeneration. Additional genes that we discuss below are prime targets for future functional studies. Moreover, having established the efficacy of a chemical perturbation approach, we note key findings that validate the axolotl embryo model for epigenetic studies of tail regeneration. While our discussion focuses on the effect of romidepsin on histone acetylation, we note that changes in transcription could reflect indirect effects of romidepsin (Li et al., 2020). For example, non-histone proteins that are normally de-acetylated and inactive during regeneration could potentially be activated by romidepsin to regulate transcription and cellular level processes.

**FIGURE 8:**
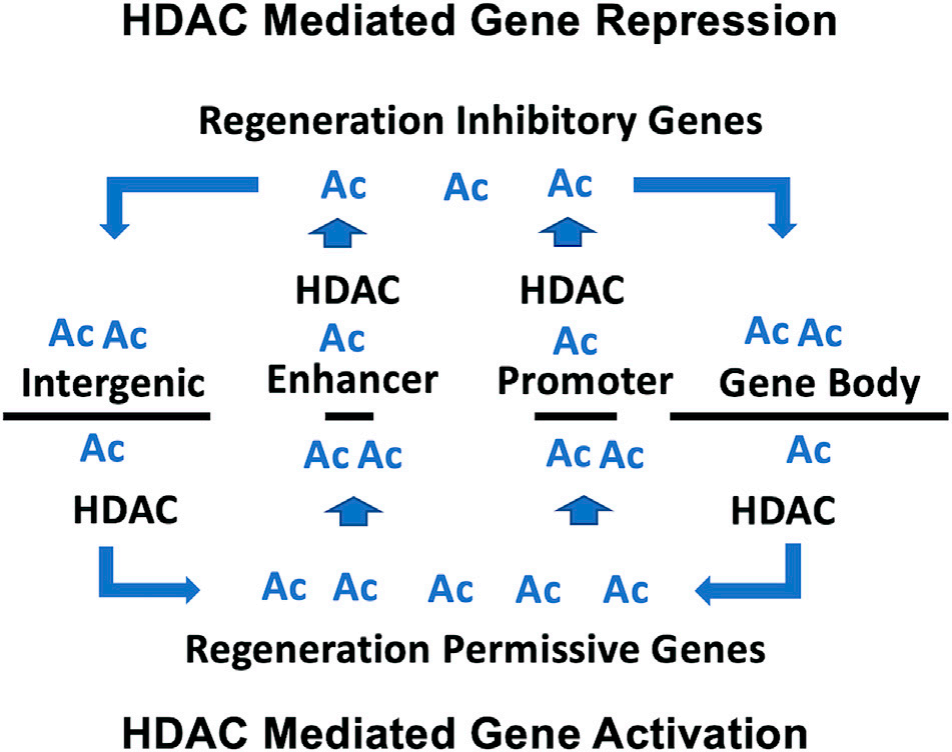
Model proposed for HDAC-mediated transcriptional regulation during tail regeneration. For HDAC mediated gene repression, HDAC activity is associated with promotor/enhancer regions to compact chromatin and prevent accessibility of transcription promoting factors. For HDAC mediated gene activation, HDAC activity is associated with intergenic regions and gene bodies, which increases the pool of acetyl lysine residues for hyperacetylation of promotor/enhancer regions and the recruitment of transcription promoting factors.

Our previous microarray study, using a single high concentration (10 μM) of romidepsin, identified genes that were significantly up and down regulated during axolotl tail regeneration (Voss et al., 2019). In this study, we modeled transcriptional change in response to different concentrations of romidepsin to generate transcription response curves. Sigmodal response curves are often observed in drug studies. Typically, monotonically increasing and decreasing responses are observed as a function of drug concentration, although more complicated biphasic responses are also observed (Calabrese 2013). We observed sigmoidal and biphasic responses which provide new insights about the mechanistic basis of romidepsin-mediated transcriptional regulation during tail regeneration. Romidepsin inhibits the activity of class I HDACs that function in the acetylation of lysine residues, including non-histone proteins. Hyperacetylation of promotor and enhancer associated histones could potentially open chromatin that is typically maintained during regeneration in a structurally compacted, repressed transcriptional state (Sterner and Berger, 2000) (Figure 8). This could potentially explain sigmoidal transcription responses for genes that were upregulated by high concentrations of romidepsin, for example *Cited2*, which is strongly downregulated after tail amputation under control conditions, implicating *Cited2* as an HDAC-regulated locus (Voss et al., 2019). In a more recent experiment, we showed that *Cited2* was more strongly upregulated when embryos were co-treated with romidepsin and cobalt chloride, a chemical that induces oxidative cellular stress in axolotl embryos (Baddar et al., 2021). As a transcriptional co-activator, *Cited2* may interact with transcription factors to induce cellular stress pathways that are inhibitory to regeneration. Indeed, there is growing appreciation for the idea that cellular immune responses must be spatially and temporally regulated after injury to ensure a successful regeneration outcome (Godwin et al., 2017). The regulation of *Cited2* transcription at the time of injury may affect cells that can plastically express stress or reparative phenotypes. Our results suggest that histone acetylation factors strongly in the regulation of *Cited2,* a hypothesis that can be tested by quantifying *Cited2* histone acetylation in romidepsin treated and untreated embryos. In addition to *Cited2* and other genes that are implicated in cell cycle arrest (*G0s2*) and negative regulation of cell signaling (*Spry1, Smad7*), HDACs may also function to repress the expression of morphogenic genes during regeneration (Wang et al., 2021).

Romidepsin mediated repression of transcription may depend upon a different acetylation-associated mechanism of gene regulation. Genes that are typically upregulated during normal embryo tail regeneration are down regulated by high concentrations of romidepsin, including *Krt17, Has2* and *Lep.* Our results show that regeneration associated genes are expressed across multiple cell types, including fibroblast-like progenitor cells. For these genes, romidepsin may affect a redistribution of histone acetylation away from promoter/enhancer regions to gene bodies and intergenic regions, which in turn would redistribute epigenetic reader proteins that mediate enhancer promotor interactions and transcriptional elongation (Greer et al., 2015; Slaughter et al., 2021). Under this model, the concentration dependent effect of romidepsin on transcription would be expected to correlate with locus-specific changes in histone acetylation (Figure 8). Chip-Seq studies of histone acetylation would likely be informative using tail tissue from axolotl embryos as the genes identified at regeneration permissive and inhibitory romidepsin concentrations appear to be regulated by transcriptional mechanisms that transcend transcriptional states and cell types, at least in this regeneration model. It remains to be determined if embryo tail regeneration presents greater transcriptional plasticity than larval and adult tail regeneration. It would also be informative to use snRNA-Seq within the context of a romidepsin concentration response experiment to determine if HDAC activity can be titrated to alternatively regulate regeneration permissive vs inhibitory gene expression outcomes within and across cell types.

In summary, we dose-dependently titrated transcription and regeneration outcome using romidepsin and an axolotl tail regeneration model. Relatively high doses of romidepsin decreased the expression of regeneration associated genes and increased the expression of genes associated with regenerative failure. Using single-nuclei RNA-Seq, we showed that HDAC mediated gene regulation is a shared property of many different cell types. Our results suggest that HDAC activity plays a central and perhaps integrative role in the regulation of transcription across cell types during tissue regeneration.

## Data Availability

The datasets presented in this study can be found in online repositories. The names of the repository/repositories and accession number(s) can be found below: Gene Expression Omnibus GSE183645.

## Supplementary Material

Supplementary Figure S1 CRISPR-Cas9 editing of the Has2 locus. A. Two gRNAs (bold andunderlined) were designed to target Has2 protein coding sequence. B. PCR primers(bold and italic) flanking the gRNA target region were used to amplify DNA fragments generated for a non-injected control and 6 injected individuals. C. Electropherogram showing the DNA sequencing result for non-injected control **(top)** and injected embryo #4 **(bottom)**.

Supplementary Figure S2 HCR-FISH of uninjured and 3 HPA axolotl tail. *Brd4* expression was broadly observed throughout the uninjured and 3 HPA tail.

Supplementary Figure S3 Correlation of fold change for 29 romidepsin moderated genes at 6 HPA. Fold changes observed control 0 and 10 μM romidepsin treatments in the Nanostring experiment correlated positively with fold changes observed between the control and romidepsin 6 HPA treatments in the single nuclei RNA-Seq experiment.

Supplementary Figure S4 Heatmap showing fold change estimates between control 0 and 0.01, 0.05, 0.5, 1.0, 5.0, and 10 μM romidepsin treatments at 6 HPA. Red colors indicate genes that were expressed more highly in romidepsin treated embryos and green colors indicates genes that were expressed more highly in control embryos. Transcriptional response classifications and inflection points for sigmoidal and biphasic models are shown for each gene.

## References

[B1] Arrowsmith C. H. Bountra C. Fish P. V. Lee K. Schapira M. (2012). Epigenetic Protein Families: a New Frontier for Drug Discovery. Nat. Rev. Drug Discov. 11, 384–400. 10.1038/nrd3674 22498752

[B2] Baddar N. W. A. H. Dwaraka V. B. Ponomareva L. V. Thorson J. S. Voss S. R. (2021). Chemical Genetics of Regeneration: Contrasting Temporal Effects of CoCl 2 on Axolotl Tail Regeneration. Develop. Dyn. 250, 852–865. 10.1002/dvdy.294 PMC8917379 33410213

[B3] Benjamini Y. Hochberg Y. (1995). Controlling the False Discovery Rate: a Practical and Powerful Approach to Multiple Testing. J. R. Stat. Soc. Ser. B 57, 289–300.

[B4] Bordzilovskaya N. P. Dettlaff T. A. Duhon S. T. Malacinski G. M. (1989). “Developmental Stage Series of Axolotl Embryos,” in The Developmental Biology of the Axolotl. Editors Armstrong J. B. Malacinski G. M. (New York: Oxford University Press), 201–219.

[B5] Calabrese E. J. (2013). Biphasic Dose Responses in Biology, Toxicology and Medicine: Accounting for Their Generalizability and Quantitative Features. Environ. Pollut. 182, 452–460. 10.1016/j.envpol.2013.07.046 23992683

[B6] Camenisch T. D. Spicer A. P. Brehm-Gibson T. Biesterfeldt J. Augustine M. L. Calabro A. Jr (2000). Disruption of Hyaluronan Synthase-2 Abrogates normal Cardiac Morphogenesis and Hyaluronan-Mediated Transformation of Epithelium to Mesenchyme. J. Clin. Invest. 106, 349–360. 10.1172/jci10272 10930438 PMC314332

[B7] Contreras E. G. Gaete M. Sánchez N. Carrasco H. Larraín J. (2009). Early Requirement of Hyaluronan for Tail Regeneration inXenopustadpoles. Development 136, 2987–2996. 10.1242/dev.035501 19666825

[B9] Duerr T. J. Jeon E. K. Wells K. M. Villaneuva A. Seifert A. W. McCusker C. D. (2021). A Constitutively Expressed Fluorescence Ubiquitin Cell Cycle Indicator (FUCCI) in Axolotls for Studying Tissue Regeneration. bioRxiv. 10.1101/2021.03.30.437716 Available at: https://www.biorxiv.org/about/FAQ PMC8977096 35266986

[B10] Godwin J. W. Pinto A. R. Rosenthal N. A. (2017). Chasing the Recipe for a Pro-regenerative Immune System. Semin. Cel Develop. Biol. 61, 71–79. 10.1016/j.semcdb.2016.08.008 PMC5338634 27521522

[B11] Greer C. B. Tanaka Y. Kim Y. J. Xie P. Zhang M. Q. Park I.-H. (2015). Histone Deacetylases Positively Regulate Transcription through the Elongation Machinery. Cel Rep. 13, 1444–1455. 10.1016/j.celrep.2015.10.013 PMC4934896 26549458

[B13] Leigh N. D. Dunlap G. S. Johnson K. Mariano R. Oshiro R. Wong A. Y. (2018). Transcriptomic Landscape of the Blastema Niche in Regenerating Adult Axolotl Limbs at Single-Cell Resolution. Nat. Commun. 9, 5153. 10.1038/s41467-018-07604-0 30514844 PMC6279788

[B14] Li G. Tian Y. Zhu W.-G. (2020). The Roles of Histone Deacetylases and Their Inhibitors in Cancer Therapy. Front. Cel Dev. Biol. 8, 576946. 10.3389/fcell.2020.576946 PMC7552186 33117804

[B15] Mi H. Muruganujan A. Huang X. Ebert D. Mills C. Guo X. (2019). Protocol Update for Large-Scale Genome and Gene Function Analysis with the PANTHER Classification System (v.14.0). Nat. Protoc. 14, 703–721. 10.1038/s41596-019-0128-8 30804569 PMC6519457

[B30] Narvaez C. J. Grebenc D. Balinth S. Welsh J. E. (2020). Vitamin D regulation of HAS2, Hyaluronan Synthesis and Metabolism in Triple Negative Breast Cancer Cells. J. Steroid Biochem. Mol. Biol. 201, 105688. 10.1016/j.jsbmb.2020.105688 32360595 PMC8432753

[B16] Ouyang X. Panetta N. J. Talbott M. D. Payumo A. Y. Halluin C. Longaker M. T. (2017). Hyaluronic Acid Synthesis Is Required for Zebrafish Tail Fin Regeneration. PloS one 12, e0171898. 10.1371/journal.pone.0171898 28207787 PMC5313160

[B17] Ponomareva L. V. Athippozhy A. Thorson J. S. Voss S. R. (2015). Using *Ambystoma mexicanum* (Mexican Axolotl) Embryos, Chemical Genetics, and Microarray Analysis to Identify Signaling Pathways Associated with Tissue Regeneration. Comp. Biochem. Physiol. C Toxicol. Pharmacol. 178, 128–135. 10.1016/j.cbpc.2015.06.004 26092703 PMC4662883

[B28] Rodgers A. K. Smith J. J. Voss S. R. (2020). Identification of Immune and Non-Immune Cells in Regenerating Axolotl Limbs by Single-cell Sequencing. Exp. Cell Res. 394, 112149. 10.1016/j.yexcr.2020.112149 32562784 PMC7483677

[B18] Schenone M. Dančík V. Wagner B. K. Clemons P. A. (2013). Target Identification and Mechanism of Action in Chemical Biology and Drug Discovery. Nat. Chem. Biol. 9, 232–240. 10.1038/nchembio.1199 23508189 PMC5543995

[B19] Schindelin J. Arganda-Carreras I. Frise E. Kaynig V. Longair M. Pietzsch T. (2012). Fiji: an Open-Source Platform for Biological-Image Analysis. Nat. Methods 9, 676–682. 10.1038/nmeth.2019 22743772 PMC3855844

[B20] Slaughter M. J. Shanle E. K. Khan A. Chua K. F. Hong T. Boxer L. D. (2021). HDAC Inhibition Results in Widespread Alteration of the Histone Acetylation Landscape and BRD4 Targeting to Gene Bodies. Cel Rep. 34, 108638. 10.1016/j.celrep.2020.108638 PMC7886050 33472068

[B21] Sterner D. E. Berger S. L. (2000). Acetylation of Histones and Transcription-Related Factors. Microbiol. Mol. Biol. Rev. 64, 435–459. 10.1128/mmbr.64.2.435-459.2000 10839822 PMC98999

[B29] Trofka A. Huang B-L. Zhu J. Heinz W. F. Magidson V. Shibata Y. (2021). Genetic Basis for an Evolutionary Shift from Ancestral Preaxial to Postaxial Limb Polarity in Non-Urodele Vertebrates. Curr. Biol. 31, 4923-4934. 10.1016/j.cub.2021.09.010 34610275 PMC8612998

[B22] Ueda H. Nakajima H. Hori Y. Fujita T. Nishimura M. Goto T. (1994). FR901228, a Novel Antitumor Bicyclic Depsipeptide Produced by Chromobacterium Violaceum No. 968. I. Taxonomy, Fermentation, Isolation, Physico-Chemical and Biological Properties, and Antitumor Activity. J. Antibiot. 47, 301–310. 10.7164/antibiotics.47.301 7513682

[B23] Varshney G. K. Pei W. LaFave M. C. Idol J. Xu L. Gallardo V. (2015). High-throughput Gene Targeting and Phenotyping in Zebrafish Using CRISPR/Cas9. Genome Res. 25, 1030–1042. 10.1101/gr.186379.114 26048245 PMC4484386

[B24] Voss S. R. Ponomareva L. V. Dwaraka V. B. Pardue K. E. Baddar N. W. A. H. Rodgers A. K. (2019). HDAC Regulates Transcription at the Outset of Axolotl Tail Regeneration. Sci. Rep. 9, 6751. 10.1038/s41598-019-43230-6 31043677 PMC6494824

[B25] Wang M. H. Hsu C. L. Wu C. H. Chiou L. L. Tsai Y. T. Lee H. S. (2021). Timing Does Matter: Nerve-Mediated HDAC1 Paces the Temporal Expression of Morphogenic Genes during Axolotl Limb Regeneration. Front. Cel Dev. Biol. 9, 641987. 10.3389/fcell.2021.641987 PMC8143519 34041236

[B26] Yang Z. Yang C. Xiao L. Liao X. Lan A. Wang X. (2011). Novel Insights into the Role of HSP90 in Cytoprotection of H2S against Chemical Hypoxia-Induced Injury in H9c2 Cardiac Myocytes. Int. J. Mol. Med. 28, 397–403. 10.3892/ijmm.2011.682 21519787

[B27] Yeh J.-R. J. Crews C. M. (2003). Chemical Genetics. Develop. Cel 5, 11–19. 10.1016/s1534-5807(03)00200-4 12852848

