# Supplementary figures and images for: HDAC Inhibitor Titration of Transcription and Axolotl Tail Regeneration
Source: Front Cell Dev Biol. 2021 Dec 31;9:767377. doi: 10.3389/fcell.2021.767377 (PMC8759488; doi:10.3389/fcell.2021.767377)

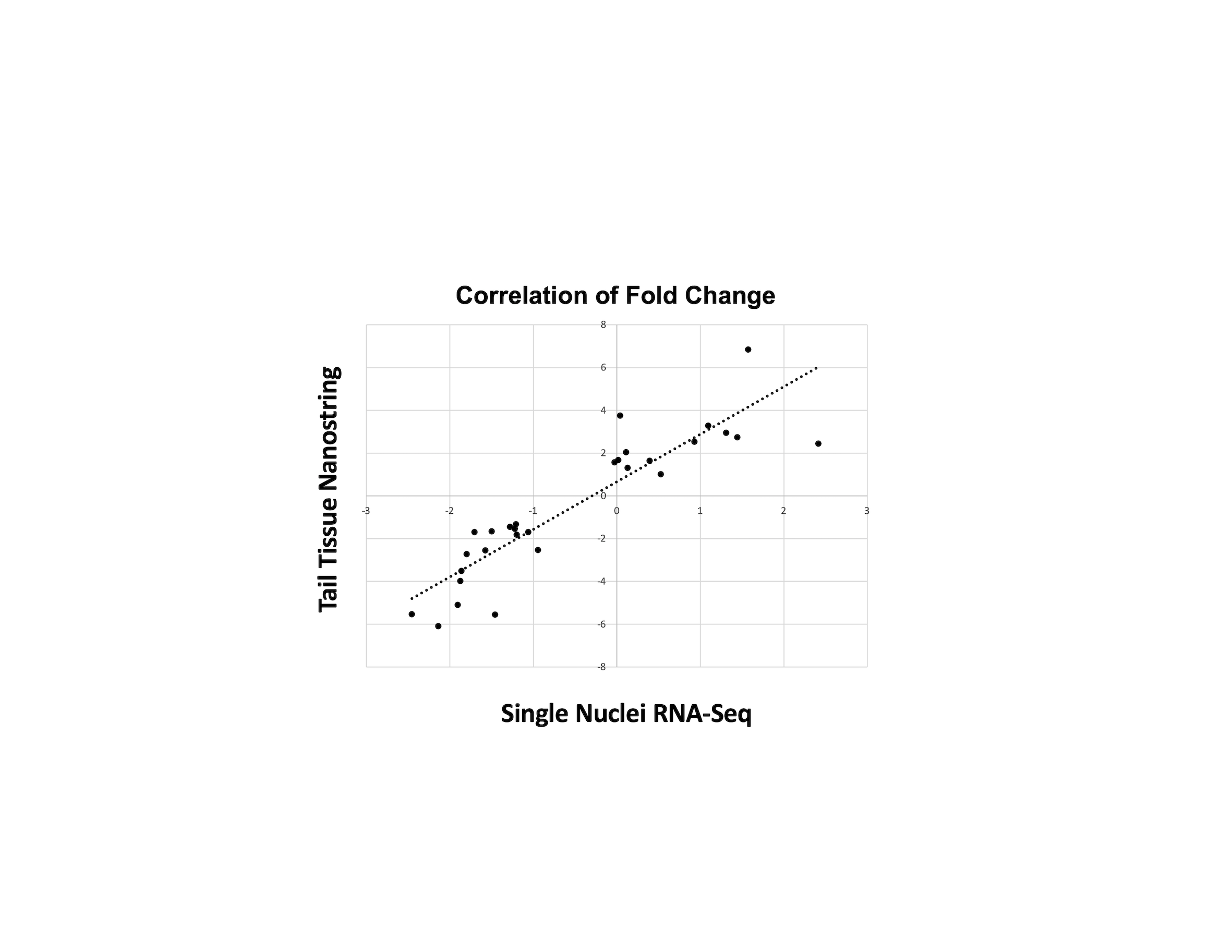

Supplement: Supplementary file 1 [file Image3.tiff]

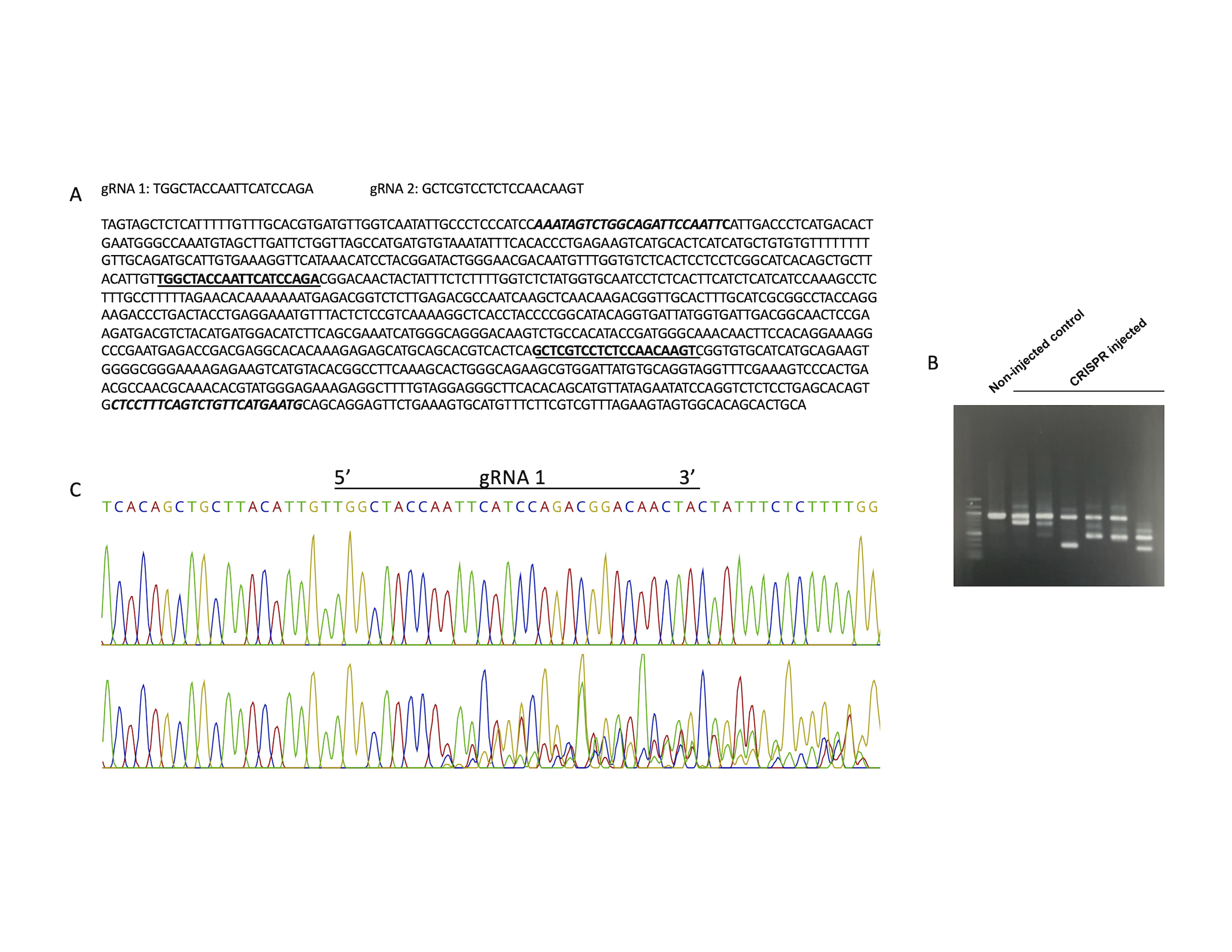

Supplement: Supplementary file 3 [file Image1.tiff]

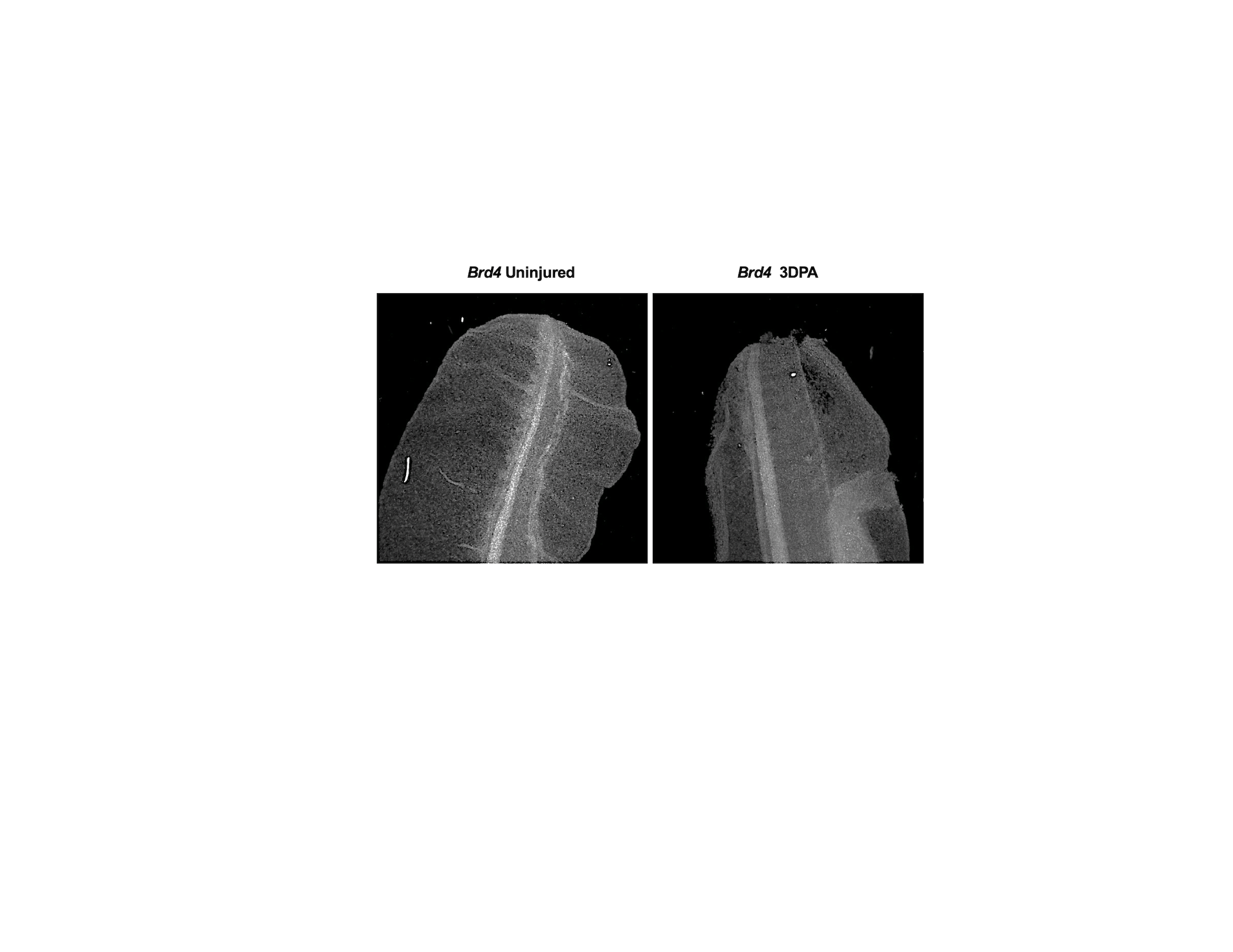

Supplement: Supplementary file 10 [file Image2.tiff]

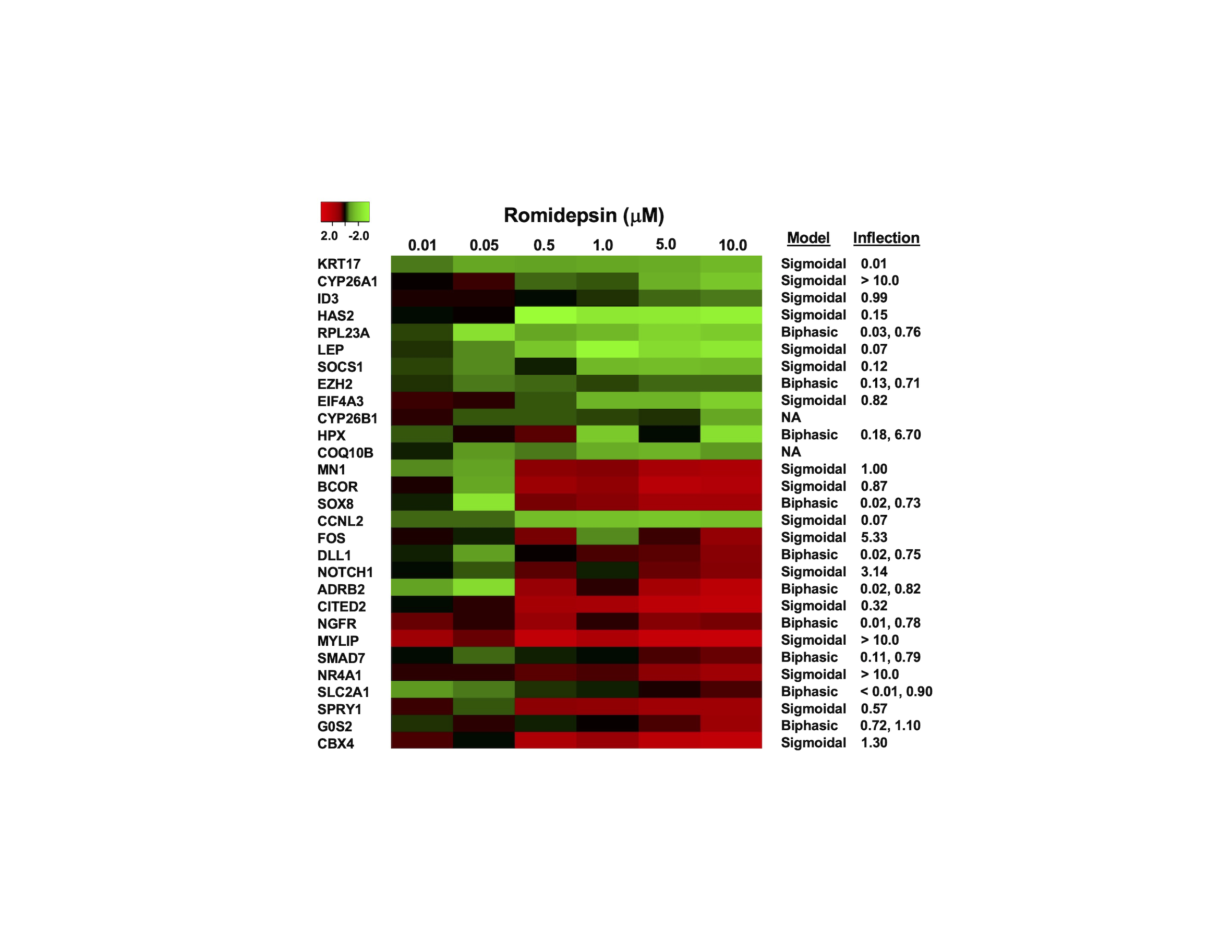

Supplement: Supplementary file 11 [file Image4.tiff]
